# Circadian rhythm disruptions: A possible link of bipolar disorder and endocrine comorbidities

**DOI:** 10.3389/fpsyt.2022.1065754

**Published:** 2023-01-05

**Authors:** Xiu Yan, Peiwei Xu, Xueli Sun

**Affiliations:** Department of Psychiatry, West China Hospital of Sichuan University, Chengdu, China

**Keywords:** circadian rhythm, bipolar disorder, endocrine comorbidities, clock gene, chronotherapy

## Abstract

Epidemiological studies have demonstrated an association between bipolar disorder (BP) and endocrine diseases. Further, circadian rhythm disruptions may be a potential common pathophysiological mechanism of both disorders. This review provides a brief overview of the molecular mechanisms of circadian rhythms, as well as roles circadian rhythms play in BP and common endocrine comorbidities such as diabetes and thyroid disease. Treatments targeting the circadian system, both pharmacological and non-pharmacological, are also discussed. The hope is to elicit new interest to the importance of circadian system in BP and offer new entry points and impetus to the development of medicine.

## Introduction

Bipolar disorder (BP) is defined as episodic elevation or decrease in mood, thinking and activity, which also includes a combination of (hypo-)manic and depressive symptoms during the same event. It is an important public health problem that affects 1.0–2.1% of the global population (1) leading to cognitive deficits, functional impairment, disability, or even death in young people (2–4). In addition to psychiatric symptoms, a significant proportion of the disease burden can be attributed to somatic comorbidities including diabetes, thyroid disease, and cardiovascular disease (5–7). Although the use of psychotropic drugs lead to metabolic risks (7–9), it is not the only cause of increased susceptibility to somatic disorders. Previous studies have reported that drug-naive BP patients still have an increased risk of metabolic related diseases (10–12). Intriguingly, patients with certain endocrine and metabolic diseases also have increased risks of mental comorbidities (13). This bidirectional association suggests a possible shared pathological pathway between BP and endocrine diseases.

Growing evidence supports the importance of circadian rhythm disruptions in the incidence and development of BP (14–16). As endogenous biological rhythms, circadian rhythms regulate physiological processes in a 24-h periodicity and synchronize them with environmental cycles, including sleep-wake cycles, endocrinology, metabolic, immunity, and blood pressure (17–21). Disturbances in circadian rhythms can lead to acute or permanent impairment of emotional, cognitive, and physical functions (22). Given the importance of circadian rhythms in physiological and pathophysiological settings, it may play a key role in the pathogenesis of BP and its endocrine comorbidities. Therefore, a better understanding of circadian rhythm may help to the management of BP and comorbidities. This review includes an overview of the molecular mechanism of circadian rhythms and its consequences for the pathogenesis of BP and common endocrine comorbidities, such as diabetes and thyroid disease. In addition, we discuss the potential clinical implications of new therapeutic possibilities that target circadian rhythm modulation for BP and endocrine comorbidities.

## Literature search strategy

A literature search was performed using PubMed/Medline and Google Scholar databases from inception to October 2022. Different combinations of the keywords BP, circadian rhythm, circadian system, clock genes, circadian genes, metabolism, endocrine diseases, diabetes, thyroid disease, metabolic diseases, comorbidity, mood stabilizers, chronobiology, and chronotherapy were polled. Only studies published in English were taken into account. Subsequently, read title and abstract for primary screening and read full-text for re-screening. We also searched relevant references according to some review articles. Identified studies were synthesized narratively to provide an overview rather than an all-inclusive systematic review.

## Molecular mechanisms of circadian rhythms

Circadian is considered as a major regulator of all aspects of human health (23). The suprachiasmatic nucleus (SCN) serves as the master circadian pacemaker, synchronizing peripheral clocks through neural and endocrine signaling mechanisms and aligning daily rhythms in physiology and behavior to day/night cycles (24). It converts light signal to rhythmic output, which drives rhythms in autonomic nervous system as well as cortisol and melatonin levels, so as to keep normal circadian periods. On the other hand, endocrine signals also influence the functioning of the molecular clock at all levels, thus adjusting internal rhythmic processes in response to changes in environmental conditions (25). This delicate balance is sensitive to external perturbations, such as diets and physical activities (26).

At the molecular level, the circadian clock is regulated by a transcriptional-translational feedback loop consisting of a set of core clock genes like CLOCK, BMAL1, PER (PER1, PER2, and PER3), and CRY (CRY1 and CRY2). CLOCK and BMAL1 heterodimerize binds to the E-box element in the promoter region of PER and CRY and promote transcription. When the translated PER and CRY proteins accumulate to a certain amount in the cytoplasm, they will form heterodimers, then enter the nucleus and inhibit the transcription activity of CLOCK/BMAL1 dimers. Through this negative feedback regulation, it can inhibit the transcription of PER and CRY genes (27). In addition, the target genes of CLOCK/BMAL1 include the nuclear receptors REV-ERB (REV-ERBα and REV-ERBβ) and ROR (RORα, RORβ, and RORγ). They competitively bind to the REV-ERB-ROR response component sites in the BMAL1 promoter regions to inhibit or activate BMAL1 transcription, respectively (23, 28). These positive/negative feedback pathways form the foundation of the molecular clock. At the post-transcriptional and post-translational levels, there are also various regulatory mechanisms involved in the generation of circadian oscillation pattern, including methylation, polyadenylation, histone modifications, and non-coding RNAs (29).

Clock and clock-controlled genes with different functions have been reported to make up 3–16% of the transcriptome in specific tissues, affecting the physiological functions of various cells and organs in a diurnal time-dependent manner. Considering that circadian regulation is an integral part of maintaining normal physiology, the disruptions of rhythms may be related with a wide range of pathological processes including mental disorders and endocrine diseases (30). As such, in the Section “Circadian rhythm disruptions in BP and endocrine comorbidities,” we elaborate existing evidence on circadian disruptions in BP and endocrine comorbidities.

## Circadian rhythm disruptions in BP and endocrine comorbidities

### Bipolar disorder and circadian rhythm disruptions

Evidence has established a genetic link between clock genes and BP, in which obvious candidates include CLOCK, ARNTL, NR1D1, PER1, PER3, CRY2, TIMELESS, and GSK3-β (31–33). Physiological markers of circadian rhythm disturbances have also been observed in patients with BP, including perturbed rhythms in melatonin, cortisol, and core body temperature (34). A periodic secretion of cortisol and melatonin is directly driven by the SCN and in turn favors maintenance of circadian rhythms. Multiple studies have proved mood state-related abnormal secretion patterns of melatonin and cortisol in BP patients, in terms of both kinetics and amount (35, 36). This endocrine imbalance, as part of desynchronization of peripheral and central clocks, may lead to defective response to physiological events that later develop into the mood episodes. Disruption of environmental cues could similarly result in the desynchronization of the internal circadian system. This was corroborated by a study which focus on how circadian rhythms and social rhythms influence each other and contribute to mood episodes (37). Taken together, the disrupted coordination among SCN, peripheral clocks and environmental variables may be an important contributor to BP could potentially be an important cause of BP.

At clinical level, BP patients display changes of rhythmicity in mood, energy, sleep, appetite, and concentration (38). As the prevalent phenotypic manifestations of circadian disruptions, sleep-wake cycle disturbances is even one of the core features of BP that run through the whole course of the disease (39). 66–99% of bipolar mania patients exhibited a decreased need for sleep, whereas those with bipolar depression reported disturbance in the form of insomnia or hypersomnia (39, 40). Additionally, 70% of BP patients suffered from continuing sleep problems even in the remission phase (40, 41). What’s more, BP patients were more prone to have late chronotype than healthy controls (14, 35). Disturbed and misaligned pattern of sleep and circadian rhythms has been found to be associated with clinical symptoms, functional deterioration, and poor outcomes (39–43). For example, the reduced circadian rhythmicity was found to be associated with longer duration of BP (43).

### Endocrine diseases and circadian rhythm disruptions

Several high-impact reviews have highlighted the role of circadian rhythm in metabolism regulation, emphasizing that its disruptions contribute to the pathogenesis of endocrine and metabolic diseases (44–46). A range of metabolic process, including glucose homeostasis, insulin sensitivity, lipid metabolism, and energy expenditure, follows a daily circadian rhythm (47, 48). Genetic studies have identified clock genes and variants were associated with metabolic syndrome, diabetes, obesity, and other human diseases (49, 50). In this process, the timing-dependent activity of the hormones is regulated by circadian rhythms, as well acting as a mediator of different parts of circadian network to in turn provide feedback information to SCN. Once this interaction is broken, various physiological disruptions may follow and increase the risk of secondary pathologies (49, 51–53). This review provides a detailed summary of diabetes and thyroid disease, since they are most frequently observed comorbidities of BP.

Diabetes, clinically characterized by hyperglycemia, is a chronic metabolic disease caused by insulin deficiency or insulin resistance. Blood glucose level in human body exhibits interesting changes in a diurnal cycle, mainly through regulation of food intake, energy expenditure, and insulin sensitivity *via* central clocks (54). Furthermore, glucose absorption, local insulin sensitivity, and insulin secretion are also regulated by peripheral clocks in intestine, muscle, adipose tissue, liver, and pancreas. Disruption of circadian rhythms would produce misalignment between behavior and light-dark cycles, and thus account for glucose homeostasis disruption and increase the risk of developing type 2 diabetes (55, 56). ARNTL and CRY2 single nucleotide polymorphisms were identified to be associated with type 2 diabetes and elevated fasting glucose, respectively (54). Dyssynchronization between different components of the circadian system and the daily rhythms of sleep-wake or food intake in humans may also lead to the development of insulin resistance (54, 57). In a behavioral cycle mismatch study, after three cycles of circadian mismatch (28 h/day), the number of rhythmic transcripts in the blood of healthy volunteers was significantly reduced, and even reported a trend toward impaired glucose tolerance and insulin resistance (18).

Thyroid hormones (TH) are important for metabolism, thermogenesis, normal growth, development and differentiation of cells and their synthesis, and secretion are mainly regulated by thyroid stimulating hormone (TSH). TSH secretion exhibits a distinct daily rhythm, controlled by SCN (57, 58). In rats that have had their pituitaries removed, TH rhythmic oscillations were eliminated, although Per1 and Bmal1 in the thyroid were still expressed in a 24-h rhythm (58). Conversely, lesions of the SCN resulted in loss of circadian rhythm of TSH and TH (59). These studies suggested that the rhythm of TH was also controlled by SCN, instead of local circadian clock in thyroid gland (58). It was observed that several thyroid diseases were associated with disrupted circadian rhythm of TSH secretion, including severe hypothyroidism, hyperthyroidism and autoimmune thyroid disease (60, 61). Moreover, a study found that malignant thyroid nodules had altered expression level of clock gene (62).

### Endocrine comorbidities of BP and circadian rhythms

Identified studies suggested that BP exhibits exceptionally high comorbidity rates with endocrine and metabolism disease, and this figure is 17% in a cross-sectional study conducting in China (63, 64). An emerging strategy to address this issue is to conceptualize BP and comorbidities as a unified whole rather than as multiple parts. Previous studies have also supported for shared pathogenic mechanisms in mental disorders and somatic diseases (5, 19, 65, 66). In the above two Sections “Endocrine diseases and circadian rhythm disruptions and Endocrine comorbidities of BP and circadian rhythms,” we can see that either BP or metabolic disease are related to circadian clock pathway. Considering that, we infer that disrupted circadian rhythms might play a crucial role in endocrine comorbidities of BP. Evidence is extensive and comes from various aspects ranging from rhythmic hormones to social rhythms.

The current meta-analysis demonstrates that hypothalamic-pituitary-adrenal (HPA) axis disturbances are present in BP patients, and considers the effect of circadian rhythm and ultradian rhythms (67). A longitudinal study revealed the potential of cortisol and gene circadian rhythm as diagnostic biomarkers and therapeutic targets in the treatment of BP (68). These activation of the HPA axis and hypercortisolemia are strongly associated with insulin resistance and hyperglycemia, which may contribute to endocrine and metabolic diseases (69). Research on sleep-related phenotypes also provides some clues. Sleep problems in BP show the correlation with cardiovascular disease, increased weight, and diabetes (41). There is also study in euthymic BD patients revealed that number of clinical diseases was independently associated with poor sleep quality (70). Macroscopically, BP appears abnormal social rhythms such as feeding and locomotor activity, which then leads to the development to aberrant metabolism (71).

## Pharmacological and non-pharmacological interventions in circadian rhythms

Given that circadian disruptions being a common pathophysiological mechanism in BP and endocrine comorbidity, approaches to normalize circadian rhythms could be potential treatment options for the integrated management. Indeed, mood stabilizers currently in use have been found to affect circadian rhythms. For example, lithium might lengthen circadian period *via* inhibition of GSK-3 and IMP, and a longer sleep-wake cycle has been observed in healthy controls clinically (36, 72–74). In contrast, valproate generally cause a shortening of circadian period by affecting the clock gene expression and hormone secretion, in both animal experiments and population-based studies (16, 75–78). In agree with our theory, a large retrospective study found that monotherapy with mood stabilizers like lithium was associated with decreased risk of diabetes (79). Moreover, agomelatine, as a melatonin MT1 and MT2 receptor agonist, is attracting growing interests in the field due to its modulation of circadian rhythms (16, 80–82). There have also been some attempts to develop new pharmacological interventions targeting clock genes. Previous studies have shown that REV-ERBα and REV-ERBβ are not only key components for molecular clock, but are also involved in the regulation of metabolism and emotion (83–85). There are some significant findings, in animal studies. REV-ERB agonist showed notable efficacy in terms of maintaining arousal, reducing anxiety, and alleviating adverse metabolic consequences of obesity, whereas REV-ERB antagonist were found to promote mania-like behavior (23). Miscellaneous agents targeting ROR, CK1, PER, and CRY also have some meaningful findings (71). For instance, stabilizer of CRY proteins could lead to circadian cycle prolongation and enhanced glucose tolerance (86). Though we do not know whether or in which conditions can these findings be kept in human studies, drugs that target circadian rhythms may have promising therapeutic potential.

Non-pharmacological interventions that target light and social rhythms are another ways to improve the stability of behavioral and biological rhythms and resynchronize the circadian rhythms (71). Such interventions include bright light therapy or dark therapy, total sleep deprivation, interpersonal and social rhythm therapy (IPSRT), and cognitive-behavioral therapy for insomnia (19, 87). Specifically, IPSRT reconstructs lifestyle regularity through interpersonal psychotherapy and behavioral therapy (88), whereas others mainly focus on sleep-wake rhythm (87). However, both the relatively small number of literatures and the lack of validated controlled design lead to a low level of evidence. A recent systematic review evaluated the efficacy of these treatments based on 19 randomized controlled studies and concluded that bright light therapy on depression was the only domain with sufficient data available for a meta-analysis (32). Non-pharmacological treatment is an important but not fully covered area, and further studies are needed to provide more evidence.

## Conclusion

Circadian rhythms broadly participate in physiological and pathological processes of human, linking BP, and endocrine comorbidities (89). This article reviews the molecular mechanisms of circadian rhythms, circadian rhythm disruptions in BP and endocrine comorbidities, and therapeutic potential for interventions targeting circadian rhythms. Somatic comorbidity causes a high disease burden for BP, treating BP, and comorbidities in parallel will bring patients more benefit. Though pharmacological and non-pharmacological interventions appear to show beneficial effects in chronobiology, many gaps remain in our understanding of the relationship between circadian clock and human health. Future studies could collect detailed data with a wider spectrum of circadian rhythms from more individuals to determine inter-individual variation of circadian rhythms and its impact on clinical status, exploring other factors which contribute to differences in individual metabolism and behavioral response. Ultimately, we hope to generate interest in the importance of the human circadian system in all aspects related to health and to bring new perspectives and entry points to the development of medicine.

## Author contributions

XY: writing – review and editing. PX: writing – original draft. XS: conceptualization. All authors contributed to the article and approved the submitted version.
